# Lower pole anatomy of horseshoe kidney and complete ureteral duplication: Anatomic and radiologic study applied to endourology

**DOI:** 10.1590/S1677-5538.IBJU.2022.99.12

**Published:** 2022-03-14

**Authors:** Ulisses Gomes, Francisco J. B. Sampaio, Luciano A. Favorito

**Affiliations:** 1 Universidade do Estado do Rio de Janeiro Unidade de Pesquisa Urogenital Rio de Janeiro RJ Brasil Unidade de Pesquisa Urogenital, Universidade do Estado do Rio de Janeiro – UERJ, Rio de Janeiro, RJ, Brasil

**Keywords:** Fused Kidney, Ureteroscopy, Anatomy

## Abstract

**Purpose:**

To analyze the 3-dimensional intrarenal anatomy of horseshoe kidneys (HK) and kidney with complete ureteral duplication (CUD), in polyester resin endocasts of the collecting system and in patients submitted to 3D computerized tomography scan (CT-scan).

**Materials and Methods:**

We analyzed seven 3-dimensional polyester resin endocasts of the kidney collecting system obtained from 6 fresh adult cadavers (4 with unilateral CUD and 2 with horseshoe kidney) and CT-scan reconstruction images of kidneys from 24 patients: 6 patients with HK, 8 with CUD and 10 patients without renal anomalies that were used as controls. We analyzed the spatial distribution of the calices, the infundibula diameters, the angle between the lower infundibulum and the renal pelvis (LIP) and the angle between the lower infundibulum and the inferior minor calyces (LIICA). Measurements of the width and length of the inferior infundibulum and the infundibula of the minor calyces, as well as the angles (LIP and LIICA) were made with the aid of the LibreOffice 6.3 software. The data were analyzed with the IBM^®^ SPSS^®^ Statistics.

**Results:**

There was no statistical difference in the inferior pole measurements between the groups with anomalies and the control group, both in polyester resin endocasts and CT-scan reconstruction images for LIP. When we compared the LIP in the CT-scan between HK versus CUD (p= 0.003), and HK versus the control group (p= 0.035), we observed statistical difference.

**Conclusions:**

The knowledge of spatial anatomy of lower pole is of utmost importance during endourologic procedures in patients with kidney anomalies. In the present study we observed that horseshoe kidneys had more restrictive anatomic factors in lower pole than the complete ureteral duplication.

## INTRODUCTION

The success rate of the treatment of calculi located in the kidney lower pole, regardless of the method used, is directly related to the anatomical parameters of this region (1, 2). Knowledge of the renal collecting system anatomy and radiological analysis of urinary system is necessary for safe and successful performance of endourological procedures (2, 3).

Calculi in the lower pole of the kidney can be treated with extracorporeal shock wave lithotripsy (SWL); retrograde flexible ureteroscopy (URS) and percutaneous nephrolithotripsy (PNL) (1). Sampaio (4) showed restrictive factors for the elimination of fragments after performing SWL (multiple calyces in the lower pole; diameter of the inferior infundibulum smaller than 04 mm and presence of angle between the lower infundibulum and renal pelvis (LIP) of less than 90°).

Horseshoe kidney is the most common of all renal fusion anomalies with a prevalence of 0.25% in the population (5, 6), and the incidence is about 1/666 births (7, 8). Ureteral anomalies of number are also frequent, with emphasis on ureteral duplications that have an incidence around 1/150 births (9). The incidence of nephrolithiasis in patients with horseshoe kidney is approximately 20% (8).

Endourologic procedures in patients with urogenital anomalies are more difficult, especially regarding the position of the renal calyces (5, 6). The knowledge of the intrarenal anatomy in these patients is important for the indication, programming and adequate performing of procedures (5-7).

There are several studies in the literature about endourologic procedures in patients with urinary system anomalies (10-12). However, studies of the intrarenal anatomy with endocasts, in cases of urinary anomalies, are rare or non-existent. The hypothesis stated in our study is that the anomalous kidneys had more restrictive anatomic factors to elimination of fragments and accessibility of URS than the normal ones.

The objective of the present study is to analyze the three-dimensional intrarenal anatomy of kidneys with congenital anomalies (horseshoe kidney and complete ureteral duplication), including: spatial anatomy of the lower pole calyces, angle between the renal pelvis and the inferior infundibulum, angle between the inferior infundibulum and the minor calyces, and width and length of inferior infundibulum, in human kidneys polyester resin endocasts and in patients submitted to abdominal 3D computerized tomography scan.

## MATERIAL AND METHODS

The present work received institutional review committee approval. This study was carried out in accordance with the ethical standards of the hospital's institutional committee on human experimentation. IRB= 4.492.916.

We analyzed 20 three-dimensional (3D) polyester resin endocasts of the kidney collecting system from our research unit. Among then, three were horseshoe kidneys (HK), seven had complete ureteral duplication (CUD) and ten 3D endocasts of kidneys without macroscopic anomalies (Control group - CG). We also analyzed 24 patients with 3D computerized tomography scan (CT-scan) reconstruction images of kidneys; among then, six were patients with HK, and eight had complete CUD kidneys; also, CT scans of 10 patients without kidney anomalies were used as CG.

The endocasts were obtained according to the technique previously described (2, 3, 13). The ureters were dissected and a yellow polyester resin was injected into the ureter to fill the kidney collecting system. Added to the resin was a styrene monomer as a diluent and a methyl ethyl ketone peroxide as a catalyst. For each 100mL of resin 10mL of styrene monomer were added, and also 3mL of catalyst and 2mL of the yellow pigment. After the injected resin had set, the kidneys were immersed in hydrochloric acid until total corrosion of the organic matter was achieved and the endocast was obtained.

**Figure 1:**
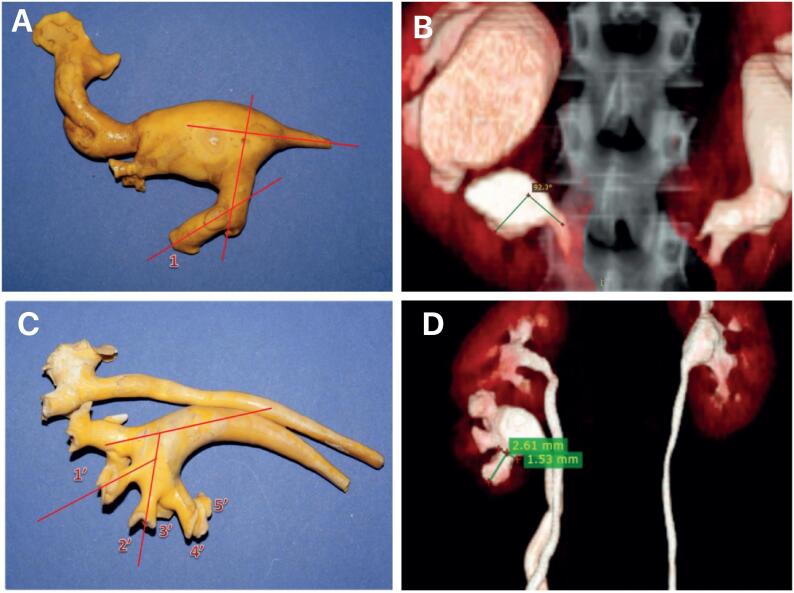
Example of the measurements performed in this study with a computer program (15). A) The figure shows an endocast of a horseshoe kidney. The angles between the lower infundibulum and renal pelvis (LIP) are measured; B) The figure shows the computed tomography (CT) scan image of one of the horseshoe kidneys analyzed showing the measurement of the LIP; C) The figure shows an endocast of a Complete Ureteral Duplication (CUD) showing the angles measurements and D) The figure shows the CT scan image of one of the CUD analyzed showing the measurement of the length and width of the infundibulum of the lower unit.

Thus, the endocasts were analyzed considering the calyceal spatial distribution and the infundibulum diameters. In the cases of duplicated pelvicalyceal collecting system we analyzed separately the superior and the inferior units. Measurements of width and length of the inferior infundibulum and infundibulum of the minor calyces and the angles were made with the aid of the LibreOffice 6.3 software (15-17), as shown in Figure-1.

The measurements on the 3D CT-scan reconstructed images were made with the Horos Project^®^ program. In the inferior pole we studied the following parameters: (a) number of major and minor calyces; (b) width and length of the infundibulum and the minor calyces; (c) angle between the lower infundibulum and renal pelvis (LIP), measured by Sampaio's (4) and Elbahnasy's methods (14); and (d) angle between the lower infundibulum and the inferior minor calyces (LIICA) (Figure-1).

### Statistical Analysis

Mean averages were statistically compared using the ANOVA with the Kruskal-Wallis test, Tukey-Krammer test and Unpaired t test (p-value < 0.05 was considered statistically significant). The data were analyzed with the IBM^®^ SPSS^®^ Statistics.

## RESULTS

The anatomical characteristics of the inferior pole analyzed in horseshoe kidneys, CUD kidneys and in the control groups, including measurements of angles, number of calyces and infundibulum measurements of the endocasts are shown in Table-1. The anatomical characteristics and measurements of the three groups studied with the 3D CT-scan are shown in Table-2. In all the 10 cases of anomalous kidneys studied we observed that the calyces are disposed in varied positions (superimposed or alternately distributed) in relation to the lateral margin of the kidney.

**Table 1:** The table shows the number of major calyces (MC) and minor calyces (mc), the mean of measurements of angles, and infundibulum of lower pole (major and minor calyces) in the sample of polyester resin endocasts of the kidney collecting system: 3 horseshoe kidneys, 7 kidneys with complete ureteral duplication (CUD) and 10 kidneys without congenital anomalies. The measurements of calyces are in millimeters (mm).

	Major Calyces (MC)	Mean Length MC ± SD	Mean Width MC ± SD	LIP ± sd	Minor Calyces (MC) ± SD	LIICA ± SD	Mean Length MC ± SD	Mean Width MC ± SD
**Horseshoe Kidney**	1 (1-1) ± 0	10.41 (6.80-13.66) ± 3.44	9.34 (5.78-11.39) ± 3.09	71.67° (16-106°) ± 48.64	3.33 (2-4) ± 1.15	42.67° (13-87°) ± 11.68	9.16 (1.95-21.25) ± 4.83	6.54 (3.24-10.25) ± 2.16
**CUD**	1 (1-1) ± 0	9.53 (5.99-12.37) ± 2.59	8.02 (4.25-13.19) ± 2.7	104.93° (70-115°) ± 9.33	2.43 (2-5) ± 1.13	36.36° (5-85°) ± 17.5	7.49 (1.95-21.25) ± 4.01	4.87 (3.24-10.25) ± 1.8
**Control Group**	1 (1-1) ± 0	12.86 (9.70-18.40) ± 3.12	6.37 (3.59-9.08) ± 1.82	92.8° (54-159°) ± 25.71	3.3 (2-5) ± 0.95	41.38° (2-139°) ± 10.71	8.95 (4.50-20.17) ± 2.1	4.96 (2.31-9.61) ± 0.98
**p value**	-	0.094	0.134	0.3003	0.227	0.0149	0.624	0.245

**LIP** = Angle between the lower infundibulum and renal pelvis; **LIICA** = angle between the lower infundibulum and the inferior minor calices. **SD** = standard deviation.

**Table 2:** The table shows the number of major calyces (MC) and minor calyces (mc), the mean of measurements of angles, and infundibulum of lower pole (major and minor calyces) in the sample of Tridimensional Computerized Tomography scan (3D CT-scan) reconstruction images of kidneys: 6 horseshoe kidneys, 8 kidneys with Complete Ureteral Duplication (CUD) and 10 kidneys without congenital anomalies. The measurements of calyces are in millimeters (mm).

	Major Calyces (MC)	Mean Length MC ± SD	Mean Width MC ± SD	LIP ± SD	Minor Calyces (MC) ± SD	LIICA ± SD	Mean Length MC ± SD	Mean Width MC ± SD
**Horseshoe Kidney**	1.33 (1-2) ± 0.52	10.17 (6.28-16.8) ± 3.64	10.51 (4.99-15.28) ± 3.88	44.97° (23.57-85.16°) ± 21.91	1 (0-2) ± 1.09	132.99° (104.82-154.67°) ± 20.7	11.69 (5.43-16.3) ± 4.2	6.86 (2.55-9.2) ± 1.86
**CUD**	1 (1-1) ± 0	15.71 (7.3-25.03) ± 6.45	9.68 (5.37-15.7) ± 3.22	86.92° (44.38-103.57°) ± 12.65	1.37 (0-3) ± 1.19	110.24° (12-170.12°) ± 53.68	7.62 (3.5-10.52) ± 2.32	8.04 (5.37-15.7) ± 3.53
**Control Group**	1.1 (1-2) ± 0.32	14.54 (1.98-22.52) ± 7.39	8.31 (3.43-15.74) ± 3.85	73.95° (47.4-114.27°) ± 24.99	1.8 (0-3) ± 1.13	136.01° (106.88-171.61°) ± 12.76	9.5 (4.14-18.89) ± 1.87	5.29 (1.65-8.54) ± 2.06
**p value**	0.180	0.267	0.491	0.004	0.403	0.382	0.119	0.205

**LIP** = Angle between the lower infundibulum and renal pelvis; **LIICA** = angle between the lower infundibulum and the inferior minor calices; **SD** = standard deviation.

### Horseshoe Kidney

The three horseshoe kidneys had the renal pelvis elongated and in a more anterior position. We observed 1 major calyx in the superior pole in the 3 cases; in the mid-kidney we observed 0 to 1 major calyx, and in the inferior pole only 1 major calyx in the 3 cases (Length: 6.8 - 13.66mm, mean=10.41mm, SD=3.44 and width=5.78 to 11.39mm, mean=9.34mm, SD=3.09). The number of minor calyces in the superior pole was 2 to 4, in the mid- kidney was 2 to 3 and in the inferior pole was 2 to 4 minor calyces (Length: 1.95 to 21.25mm, mean=9.16mm, SD=4.83 and width: 3.24 to 10.25mm, mean=6.54mm, SD=2.16). The LIP was between 16° and 106° (mean=71.67°, SD=48.64). The LIICA was between 13° and 87° (mean=42.67, SD=11.68).

In the sample of 3D CT-scan horseshoe kidneys, we analyzed 6 units. They followed the pattern of the endocasts, with elongated and more anterior position of renal pelvis. We observed 1 major calyx in the superior pole in all 6 cases; in the mid-kidney we observed 2 to 4 major calyx and in the inferior pole 1 to 2 (Length: 6.28 to 16.8mm, mean = 10.17, SD= 3.64 and width: 4.99 to 15.28mm, mean=10.51, SD=3.88). The number of minor calyces in the superior pole was 0 to 5; in the mid-kidney we also observed 0 to 5, and in the inferior pole we observed 0 to 2 (length: 5.43 to 16.3mm, mean=11.69, SD=4.2 and width: 2.55 to 9.2mm, mean=6.86, SD=1.86). The LIP was between 23.57° to 85.16° (mean=44.97, SD=21.91) and LIICA was 104.82° to 154.67° (mean=132.99, SD=20.7).

### Complete Ureteral Duplication Kidneys

In the 7 endocasts with CUD, in the superior pole we observed 1 to 2 major calyces; the number of minor calyces varied from 3 to 5. In the mid-kidney, the number of major calyces was 1 to 3. In the inferior pole we observed only 1 major calyx in all cases (Length: 5.99 to 12.37mm, mean=9.53mm, SD=2.59 and width: 4.25 to 13.19mm, mean=8.02, SD=2.7). The number of minor calyces in the inferior pole was 2 to 5 (length: 1.95 to 21.25mm, mean=7.49, SD=4.01 and width: 3.24 to 10.25mm, mean= 4.87, SD= 1.8). The LIP was between 70° and 115° (mean=104.93°, SD=9.33). The LIICA was between 5° and 85° (mean=36.36°, SD=17.5).

In the group of 8 patients with CUD analyzed with 3D CT-scan we observed in the superior pole 1 to 2 major calyces; the number of minor calyces varied from 0 to 5. In the mid-kidney, the number of major calyces was 1 to 6. In the inferior pole, we observed 1 major calyx in all cases (length: 7.3 to 25.03mm, mean=15.71, SD=6.45 and width: 5.37 to 15.7mm, mean=9.68, SD=3.22). The number of minor calyces in the inferior pole was 0 to 3 (length: 3.5 to 10.52mm, mean=7.62, SD=2.32 and width: 3.9 to 14.27mm, mean= 8.04, SD=3.53). The LIP was between 44.38° to 103.57° (mean= 86.92°, SD= 12.65) and LIICA was between 12° to 170.12° (mean=110.24°, SD= 53.68).

### Control Group

In 10 endocasts of control group we observed in the superior pole, 1 major calyx in all 10 kidneys. The mid-kidney, had 0 to 3. In the inferior pole we observed 1 major calyx in all 10 kidneys (Length: 9.7 to 18.4mm, mean=12.86mm, SD=3.12 and width= 3.59 to 9.08mm, mean=6.37mm, SD=1.82). The number of minor calyces in the inferior pole was 2 to 5 (Length: 4.5 to 20.17mm, mean=8.95mm, SD=2.1 and width: 2.31 to 9.61mm, mean=4.96mm, SD=0.98). The LIP was between 54 and 159° (mean=92.8°, SD=25.71). The LIICA was between 2° and 139° (mean=41.38°, SD=10.71).

In the 10 patients of control group studied with 3D CT-scan we observed in the superior pole 1 to 3 major calyces. The mid-kidney had 1 to 4 major calyces. In the inferior pole we observed 1 to 2 major calyces (length: 1.98 to 22.52mm, mean= 14.54, SD=7.39 and width: 3.43 to 15.74mm, mean=8.31, SD=3.85). The number of minor calyces in the inferior pole was 0 to 3 (length: 4.14 to 18.89mm, mean=9.5, SD=1.87 and width: 1.65 to 8.54mm, mean=5.29, SD=2.06). The LIP was 47.4° to 114.27° (mean= 73.95, SD=24.99) and LIICA was 106.88° to 171.61° (mean=136.01, SD=12.76).

There was no statistical difference in the measurements of the inferior pole (length and width of major and minor calyces, and LIICA) between the groups with anomalies and the control group, both in polyester resin endocasts and in 3D CT-scan reconstruction images, as well for the LIP in the resin endocasts. We observed statistical difference when we compared the LIP in the 3D CT-scan between HK versus CUD (p= 0.003) and HK versus CG (p= 0.035). When we compared polyester resin endocasts versus 3D CT-scan reconstruction images, analyzing only the lower pole, we observed statistical difference: in LIP (p= 0.006), LIICA (p= < 0.001) and length of major calyces between CUD groups (0.034).

## DISCUSSION

The anomalies of the urinary system are frequent and correspond to one third of all congenital malformations (1, 6). Congenital anomalies of the upper urinary tract comprise a diversity of abnormalities, including aberrant location, orientation and shape of the kidney, as well as variation of the collecting system and blood supply (6). The anatomic properties of anomalous kidneys present substantial obstacles to endourological procedures as consequence of the anatomical alterations, in special the position of the renal calices (18-20).

During embryogenesis, the normal ascent of the kidneys is stopped by the fusion of the lower poles, resulting in incomplete rotation, determining an anterior position of the collecting system. The horseshoe kidney has the insertion of ureter into the renal pelvis, superiorly and laterally displaced, leading to an impaired drainage of the collecting system, which predispose the patient to urinary tract infection (UTI) and urolithiasis, with an incidence of 21% to 60% (7). The anterior position of the horseshoe kidney pelvis, the varied positions of the calyx in relation to the kidney lateral margin, and the infundibula diameters (specially, in the inferior pole) are important anatomical features to be considered in patients with upper tract anomalies before endourologic procedures (6, 7).

Ureteral duplication may be incomplete or complete. If there are two separate pelvicalyceal systems joining at the ureteropelvic junction (UPJ), it is considered a bifid pelvis. On the other hand, if there are two separate ureters at the proximal aspect and they join at any point below the UPJ, before entering the bladder, the patient is considered to have bifid ureters (21).

A recent paper shows that due to the position and structure of the horseshoe kidney, the flexible ureteroscope needs to stay in large deflective status for relatively long time during the operation but remains effective in the resolution of moderate stone size in patients with horseshoe kidneys (22). LIP is one of the most important factors for successful results, although there is controversy about the limit considered unfavorable, varying from <30° to <90°, depending on the study (14, 23-25). According to Elbahnasy (14) the LIP>70° are considered a favorable factor to eliminate calculi from the lower pole.

In our sample, we observed that in the 3 endocasts of horseshoes kidneys studied, there were no differences in LIP when compared to controls. In HK endocasts the LIP has a mean of 71.67°, nevertheless, in one case (33.33%), the LIP was 16°, a very restrictive condition to ureteroscope access. In reconstructed 3D CT-scan images of horseshoe kidneys, we observed a mean of 44° in LIP with significant difference when compared to control group, nevertheless, in 5 CT-scan HK (83.3%) the LIP was lower than 70° and in one case (16.6%) the LIP was 23°, an unfavorable factor to ureteroscope access, as well as to eliminate calculi fragments.

In endocasts of kidney with CUD the LIP mean was 98.50° without differences when compared to controls, also, we did not observe cases of restrictive factors to ureteroscopic access. In 3D CT-scan of CUD the LIP mean was 88°, without differences when compared to controls, nevertheless, in 5 CUD studied (50%) the LIP was lower than 70°, and in 1 case the angle was 44°, unfavorable to uereteroscopic access.

Size and volume of calices are also limiting factors for URS success, regardless of location (26). Long infundibular length (> 3cm) and narrow width (< 5mm) lead to lower URS success rates (14). In our sample we did not observe differences in the measurements of length and width of major and minor calyces, and LIICA of inferior pole between the groups of anomalies and control group in polyester resin endocasts and 3D CT-scan reconstruction images. In CUD and horseshoe kidneys analyzed we did not observe limiting factor in lower pole regarding the size of calices, which could lead to additional difficulties during endourologic procedures (4).

When we compared polyester resin endocasts versus 3D CT-scan reconstruction images, we observed some statistical differences in measurement of lower pole highlighting the LIP (p= 0.006), the LIICA (p= < 0.001) and the length of major calyces between CUD groups (0.034). This find is very interesting and important and raises an important question: which of the measures taken is more reliable? Endocasts or 3D? The study with 3D CT-scan is more feasible but new studies with bigger samples will be necessary to answer this question.

For our surprise, we observed significant differences only in LIP of horsehoe kidney in comparation to the control group; we did not find differences in other anatomic factors. In CUD we did not observe significant differences in lower pole anatomy compared with control group, and this is a very interesting point. Some recent papers show good results with URS in horseshoe kidneys (22) and some papers showed that the PNL presented more difficulties to treat the stones in HK anomaly (less stone free, more transfusion) (7). The anatomic findings of the present paper shows that the horseshoe kidneys had more restrictive factors than the CUD in lower pole. Ectopic kidneys show great variation in origin, number, and size of renal arteries and veins (5). The complications after PNL in patients with horseshoes kidney could be more consistently explained by vascular anomalies instead of collecting system restrictive factors, nevertheless, in general, the presence of a horseshoe kidney does not affect the outcome of PNL and URS (27).

We have to mention some limitations of this study: a) the small sample size. It is important to note that the access to human kidneys to endocast obtainment is very limited; horseshoe kidney and complete ureteral duplication are very rare, so observations on a small sample may be important, although the small number is a weakness; b) it would be interesting to see if there is a correlation between our postmortem measurements and measurements taken by MRI, CT or US (either 2-D or 3-D), but due to technical difficulties this evaluation was not possible in our sample. The major limitation of the study was the impossibility of having previously performed URS and SWL in endocasts and in 3D-CT scan patients in order to confirm the caliceal accessibility with a flexible ureteroscope.

## CONCLUSIONS

The spatial anatomy of lower pole is of utmost importance during endourologic procedures in patients with kidney anomalies. In the present paper we observed that horseshoe kidneys had more restrictive anatomic factors in lower pole than the complete ureteral duplication.

## ABBREVIATIONS

3D: three-dimensional
CG: Control group - CG
CUD: complete ureteral duplication
HK: horseshoe kidneys
LIICA: angle between the lower infundibulum and the inferior minor calyces
LIP: angle between the lower infundibulum and renal pelvis
PNL: Percutaneous Nephrolithotripsy
SWL: Extracorporeal Shock Wave Lithotripsy
URS: Retrograde Flexible Ureteroscopy
